# Regional Arterial Infusion Chemotherapy improves the Pathological Response rate for advanced gastric cancer with Short-term Neoadjuvant Chemotherapy

**DOI:** 10.1038/srep17516

**Published:** 2015-12-01

**Authors:** Zhen-Feng Wu, Qin-Hong Cao, Xiao-Yu Wu, Che Chen, Zhe Xu, Wei-Su Li, Xue-Quan Yao, Fu-Kun Liu

**Affiliations:** 1 Department of Surgical Oncology, Jiangsu Province Hospital of Traditional Chinese Medicine, The Affiliated Hospital of Nanjing University of Chinese Medicine, Nanjing 210029, China

## Abstract

To identify clinicopathologic and treatment variables that could predict pathologic tumor response to short-term neoadjuvant chemotherapy (NAC) for patients with locally advanced gastric cancer. A retrospective analysis was conducted of 178 patients who underwent short-term NAC with EOX regimen followed by surgery from January 2008 to December 2010. Neoadjuvant treatment response was evaluated using tumor regression grade. Relationships between pathologic tumor response and clinicopathological factors were evaluated using logistic regression analysis. The benefits of regional arterial infusion chemotherapy were investigated separately. The postoperative pathological response rate was 46.1% (82/178) and 4 patients (2.2%) had complete pathological remission. Pathological response was significantly associated with tumor differentiation (P = 0.008), abnormal a-fetoprotein levels (P = 0.01) and administration approach to chemotherapy (intravenous versus regional arterial infusion chemotherapy) (P = 0.018). Most bone marrow toxicities, vomiting, nausea, alopecia, and fatigue were acceptable. Grade 3/4 toxicities were not commonly observed. The 3-year overall survival (OS) and recurrence free survival (RFS) were 67.0% and 53.0%, respectively. Regional arterial infusion NAC group had significantly better median RFS (48.0 versus 34.0 months) than the intravenous NAC group (P = 0.049). In conclusion, regional arterial infusion NAC can improve the pathological response rate of advanced gastric cancer treated with EOX regimen.

In China, gastric cancer is the third most common cancer but the leading cause of all cancer related deaths^1^. Surgical resection is considered the mainstay of therapy, and 30%–50% of patients can be treated operatively with curative intent. However, the majority of patients presenting with resectable gastric cancer will have locally advanced disease for which the chance of cure with surgery alone is poor^2^. Recently, the joint guidelines for the management of gastric cancer published by the three main European Oncologic Societies (ESMO, ESSO, ESTRO) properly emphasize the role of multidisciplinary treatment^3^. In particular, neoadjuvant chemotherapy (NAC) has been advocated as the preferred pathway for operable disease^3^. NAC has been shown to cause tumor downstaging and improve survival in patients with resectable locally advanced gastric cancer^4^. Response to neoadjuvant treatment is also the most important predictor of survival after curative resection of gastric cancer^5^. Although the possibility of a pathological complete response (pCR) might be increased by long-term chemotherapy, it may also increase the risk of the tumor growing and becoming unresectable. Short-term NAC is based on the following evidence-based conclusions: pCR is important to improve respectability; pCR can be achieved within two courses^6^; resectable tumors should be resected before they progress to become unresectable; and clinical response and resectability are important to improve survival^7^. However, pCR is extremely rare following short-term NAC, regardless of the regimen^8^,^9^. To improve the pathological response rate, it is important to clarify risk factors of poor pathological remission associated with short-term NAC.

With this background, the present study sought to identify clinicopathologic parameters that may predict pathologic tumor response to short-term NAC in locally advanced gastric cancer.

## Materials and Methods

### Patients

Preoperative regional arterial infusion chemotherapy has been used in advanced gastric cancer for many years in China^10^,^11^. A retrospective analysis was performed of consecutive patients with advanced but resectable gastric cancer who were admitted to Jiangsu Province Hospital of Traditional Chinese Medicine, Nanjing University of Chinese Medicine (Nanjing, China), treated in neoadjuvant intention with preoperative intravenous chemotherapy and preoperative regional arterial infusion chemotherapy between January 2008 and December 2010. They met all of the following criteria: age >20 and ≤75 years; Eastern Cooperative Oncology Group performance status (ECOG PS) ≤1; able to take oral drug; and adequate hepatic, renal, respiratory, and bone marrow function. Patients received with other regimen during the preoperative period were excluded. The selection of administration approach to NAC was not random, but was determined by surgeon’s preference and the patient’s decision. Due to economic, medicare policy, or unspecified causes, some patients did not have regional arterial infusion chemotherapy. The study was approved by the ethics committee of the Jiangsu Province Hospital of Traditional Chinese Medicine and was carried out in accordance with it. Written informed consent was obtained from the patients.

### Preoperative workup

A detailed clinical history, careful physical examination, recording of concomitant medications, assessment of performance status, hematologic and biochemical profiles, and electrocardiography were routinely performed in all patients. Initial staging included endoscopy of the upper gastrointestinal tract with multiple biopsies, computed tomography scan of the thorax and abdomen and an endoscopic ultrasound. Other diagnostics, including positron emission tomography/computed tomography or diagnostic laparotomy, were optional.

### Preoperative Chemotherapy

On day 1 of every 3-week cycle, patients treated with intravenous chemotherapy following EOX regimen received an intravenous bolus of epirubicin (at a dose of 50 mg/m^2^); oxaliplatin (at a dose of 130 mg/m^2^) was administered intravenously during a 2-hour period; oral capecitabine (625 mg/m^2^ twice daily) were given throughout treatment. Treatment cycles continued for two cycles.

Preoperative regional arterial infusion Chemotherapy following EOX regimen is as follows: on day 1, 50 mg/m^2^of epirubicin and 130 mg/m^2^of oxaliplatin, arterial interventional injection to where tumor is located; oral capecitabine (625 mg/m^2^ twice daily) were given throughout treatment. Intra-arterial infusion chemotherapy was performed via transfemoral artery route using the Seldinger’s approach before surgery. Celiac axis angiogram was initially carried out to document the visceral arterial anatomy and the arterial supply of tumor, and the digital subtraction technique was utilized in the study. According to the results of angiogram, the main blood supplying arteries of gastric cancer were detected and superselective catheterization of these arteries was performed. Then, chemotherapy drugs were administered via the placed catheter. This regimen was repeated once every 3 weeks. Treatment cycles continued for two cycles.

### Treatment Modalities

Patients proceeded to surgery during the 2–3 weeks after completion of the neoadjuvant chemotherapy. After laparotomy, the resectability was evaluated. The standard surgery for primary gastric cancer was D2 lymphadenectomy. The definition for lymphadenectomy was based with the Japanese Research Society for Gastric Cancer rules^12^. Depending on the location of the primary tumor, the surgeon performed either a total or distal subtotal gastrectomy. After a macroscopic curative resection was achieved, the patients were strongly recommended to undergo postoperative chemotherapy using intravenous chemotherapy following EOX regimen for 4 months, as long as the tumors did not recur. Pathologic analyses of the tumor specimens were performed following surgery. The pathologic treatment response was determined by two pathologists re-analyzing pathology slides. Neoadjuvant treatment response was evaluated using tumor regression grade (TRG), which was microscopically evaluated using the scale proposed by Mandard’s *et al*.^13^. Regression was graded as follows: TRG0, no regression, fibrosis was completely absent; TRG1, minor regression, dominant tumor mass with obvious fibrosis in 25% or less of the tumor mass; TRG2, moderate regression, dominant tumor mass with obvious fibrosis in 26% to 50% of the tumor mass; TRG3, good regression, dominant fibrosis outgrowing the tumor mass; more than 50% tumor regression; TRG4, total regression, no viable tumor cells; only fibrotic mass. TRG 3 and 4 was defined as tumor regression for the purpose of statistical analysis, which was referenced by previous studies^14^. Acute toxicities were graded according to the National Cancer Institute Common Toxicity Criteria version 3.0. Curative resection (R0) was defined by the absence of tumor macroscopically or microscopically after operation. The severity of surgical morbidity was evaluated by the Clavien–Dindo classification^15^.

### Follow-up

The clinical data of all patients were recorded prospectively. After gastrectomy, follow-up included routine blood tests, physical examination, and abdominal ultrasonography every 3 months postoperatively for the first 2 years and twice a year thereafter at our hospital. Recurrences were evaluated by physical examination, ultrasonic inspection, chest radiography, CT, PET-CT, MRI, endoscopy, or histological biopsy.

### Statistical analysis

Overall survival (OS) was measured from the date of surgery. Recurrence free survival (RFS) was calculated from the date of surgery to the date of the first documented clinical disease recurrence. Comparison between groups was examined with Chi-square test or Fisher’s exact test. Logistic regression analysis was used to identify correlations between pathologic response and continuous variables. OS and RFS curves were calculated using the Kaplan-Meier method. The log-rank test was used to assess survival differences. All statistical analyses in this study were performed with software package SPSS 18.0 (SPSS Inc., Chicago, IL).Statistical significance was defined as P < 0.05.

## Results

### Patients

A total of 178 patients received with EOX regimen NAC at Jiangsu Province Hospital of Traditional Chinese Medicine, Nanjing University of Chinese Medicine (Nanjing, China) from January 2008 to December 2010. The patients included were 130 males and 48 females with a median age of 64 years. Out of 178 patients, 96 patients received with intravenous NAC and 82 patients with regional arterial infusion NAC.

### Response

None of the patients had disease progression during induction chemotherapy. 82 (46.1%) patients showed tumor regression (i.e., TRG 3 or 4) as defined in the present study, and 4 patients (2.2%) had complete pathological remission. Many clinical factors were tested for their impact on achieving tumor regression such as sex, age, body mass index (BMI), serum tumor markers CEA, CA72-4, CA19-9, AFP, tumor location, tumor differentiation, tumor Borrmann type, clinical T classification, clinical N classification and administration approach to NAC. Univariate analysis revealed that tumor differentiation (P = 0.002), clinical N classification (P = 0.01), AFP (P = 0.001) and administration approach to NAC (P = 0.016) were significantly associated with pathological response (Table 1). Multivariate logistic regression analysis was performed to determine which associated factors identified by univariate analysis were independent predictive factors. The results are shown in Table 1. Tumor differentiation (hazard ratio [HR] 0.404, 95% CI 0.203–0.792, P = 0.008), AFP (HR 0.134, 95% CI 0.029–0.616, P = 0.01) and administration approach to NAC (HR 2.148, 95% CI 1.138–4.053, P = 0.018) were found to be significantly associated pathological response.

### Chemotherapy-Related Toxicities

The most frequently detected toxicities (all grades) in intravenous chemotherapy group were anorexia in 90 (93.8%), followed by nausea in 87 (90.7%), vomiting in 84 (87.5%), fatigue in 72 (75.0%), leucocytes in 64 (66.7%), and neutrophils in 59 (61.5%) (Table 2). While those in the arterial infusion chemotherapy group were nausea in 69 (84.1%), followed by anorexia in 68 (82.9%), vomiting in 65 (79.3%), fatigue in 53 (64.6%), leucocytes in 41 (50.0%), and neutrophils in 38 (46.3%) (Table 2). Most bone marrow toxicities, vomiting, nausea, alopecia, and fatigue were slightly higher, but still acceptable, in the intravenous chemotherapy group. Grade 3/4 toxicities were not frequently observed for either the arterial infusion chemotherapy group or intravenous chemotherapy group. Grade 3/4 toxicities occurred in less than 10% of patients in two groups.

### Operative outcomes

R0 resection was achieved in all patients. Out of 178 patients, 94 patients received total gastrectomy and D2 dissection and 84 patients underwent distal gastrectomy and D2 dissection. 34 patients who received D2 total gastrectomy received splenectomy.

Surgical morbidity occurred in 16 patients (9.0%). The postoperative complications (all grade) is shown in Table 3. Grade 3 morbidities included anastomotic leakage, pancreatic fistula, and postoperative bleeding. No surgical mortality was observed.

### Recurrence and Survival outcomes

**Figure 1:**
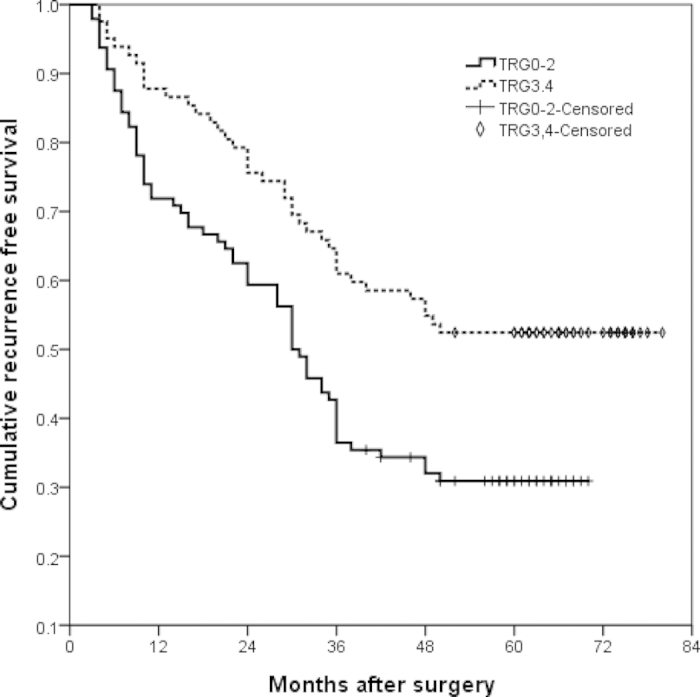
Recurrence free survival (RFS) curves of tumor regression grade (TRG) 0–2 cases and TRG3-4 cases. The 1- and 3-year RFS rate of TRG0-2 cases were 72% and 43%, and that of TRG3-4 cases were 88% and 65%, (P = 0.003).

**Figure 2:**
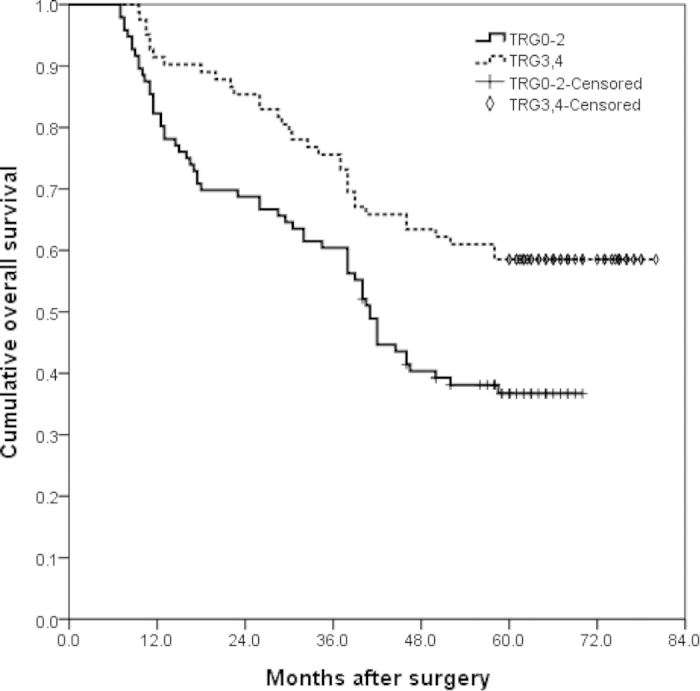
Overall survival (OS) curves of tumor regression grade (TRG) 0–2 cases and TRG3-4 cases. The 1-, 3-, and 5-year OS of TRG0-2 cases were 82%, 60% and 37%, and of TRG3-4 cases were 91%, 76% and 59%, respectively (P = 0.002).

**Figure 3:**
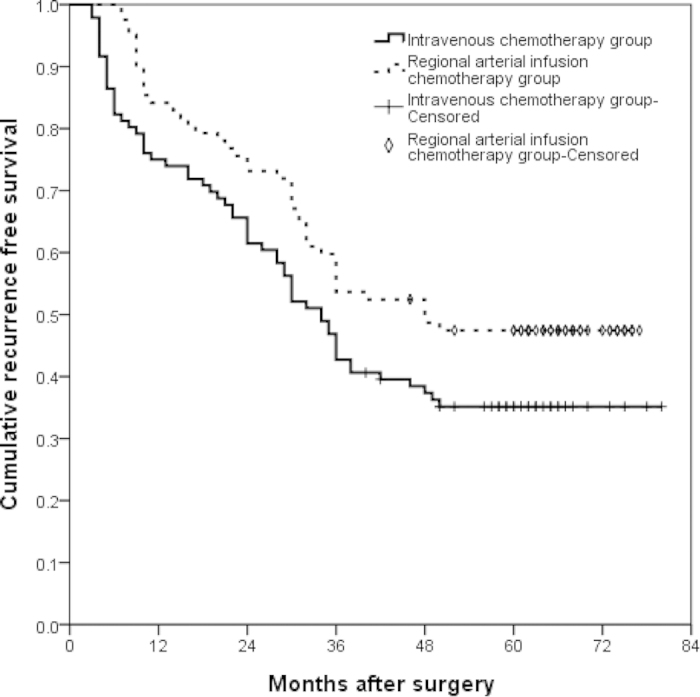
Recurrence free survival (RFS) curves of intravenous NAC group and regional arterial infusion NAC group. There were significant differences between the two groups (*P* = 0.049). The 1- and 3-year RFS rate of intravenous NAC group were 75% and 47%, whereas that of regional arterial infusion NAC group were 84% and 60%.

**Figure 4:**
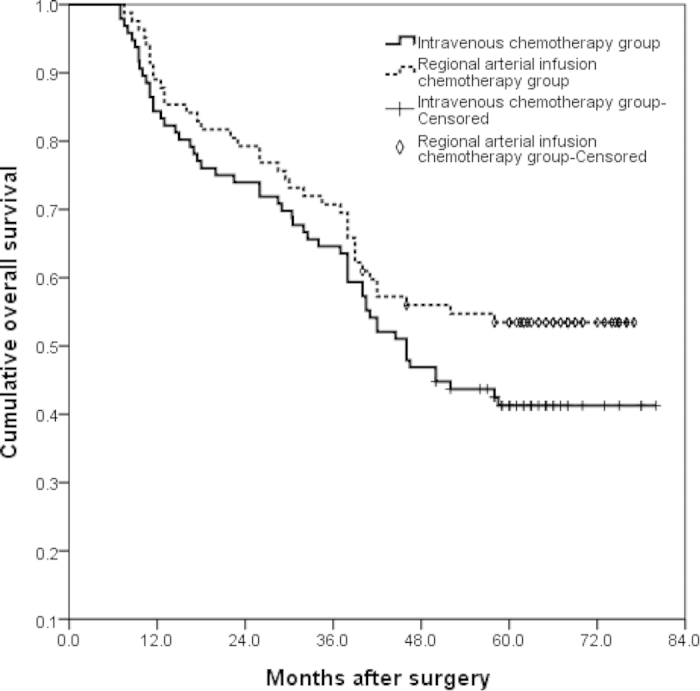
Overall survival (OS) curves of intravenous NAC group and regional arterial infusion NAC group. There was no statistically significant difference between the two groups (*P* = 0.137). The 1-, 3-, and 5-year OS rates were 84%, 65%, and 41% in the intravenous NAC group and 89%, 71%, and 53% in the regional arterial infusion NAC group.

Patients were followed up until death or until December 31, 2014. Median follow-up was 48.25 months [95% confidence interval (CI) 40.75–58.0 months]. The 1- and 3-year RFS rates were 79% and 53%, and the 1-, 3-, and 5-year OS rates were 87%, 67%, and 47%. When the survival rate was separated by TRG, the 1- and 3-year RFS rate of TRG0–2 cases were 72% and 43%, whereas that of TRG3–4 cases were 88% and 65%,(P = 0.003) (Fig. 1). The 1-, 3-, and 5-year OS of TRG0–2 cases were 82%, 60% and 37%, whereas that of TRG3–4 cases were 91%, 76% and 59%, respectively (P = 0.002) (Fig. 2). There were no significant differences in surgical and clinical pathological characteristics of patients between intravenous NAC group and regional arterial infusion NAC group. (Table 4). The RFS and OS of patients in intravenous NAC group and regional arterial infusion NAC group are shown in Figs 3 and 4. Improved RFS rates were observed in the regional arterial infusion NAC group. The 1- and 3-year RFS rate of intravenous NAC group were 75% and 47%, whereas that of regional arterial infusion NAC group were 84% and 60%, (P = 0.049) (Fig. 3). However, there was no significant difference in OS between the two groups. The 1-, 3-, and 5-year OS rates were 84%, 65%, and 41% in the intravenous NAC group and 89%, 71%, and 53% in the regional arterial infusion NAC group, respectively (P = 0.137) (Fig. 4).

## Discussion

Neoadjuvant chemotherapy (NAC) is being increasingly used in the treatment of advanced gastric cancer. Accurate evaluation of chemotherapeutic response also plays a key role in the study. The response of the primary tumor is hard to be evaluated clinically, as the primary tumor is generally a nonmeasurable lesion. Morphologic changes such as tumor size before or after chemotherapy were served as observed indicators in WHO or RECIST criteria was highly inaccurate. It was found in clinical studies that even if tumor exhibited a pathological complete response, the changes in size and shape of tumor were not very obvious^16^. The histological changes resulting from chemotherapy offers another method of evaluating tumor regression. Therefore, our study use tumor regression grade to evaluate the chemotherapeutic response and to make the results more reasonable. In the present study, postoperative pathological response rate was 46.1% (82/178) and 4 patients (2.2%) had complete pathological remission. Tumor differentiation, abnormal a-fetoprotein levels and administration approach to chemotherapy is the most important clinical predicator of pathologic tumor response.

The various histologic types of gastric cancer possess different clinical and tumor characteristics^17^. A recently study also show that better differentiated was associated with higher clinical response rate treated with XELOX regimen^18^. But Ajani *et al*. demonstrated that clinical parameters such as histological type are unable to predict tumor regression in gastric cancer patients treated with paclitaxel-based chemoradiotherapy^19^. In our study, univariate and multivariate analysis found that tumor differentiation was an important predictor of pathologic tumor regression.

A-fetoprotein (AFP) is one of the most common markers for fetal hepatoblasts, hepatic progenitor cells or hepatocellular carcinoma, whereas serum AFP of >20 ng/mL is present in approximately 1.3%–15% of gastric cancer patients^20^,^21^,^22^. Some studies reported that AFP-producing gastric cancer has been associated with advanced stage, liver metastasis, and dismal prognosis^20^,^23^,^24^. It also has been shown that AFP-producing gastric cancer had higher proliferative activity, weaker apoptosis, and richer neovascularization than that of AFP-negative gastric cancers^25^. In the present study, we find that abnormal AFP level was associated with poor pathological remission, and is the most important clinical predicator of pathologic tumor response.

ECF is routinely used in the perioperative treatment of gastric cancer^4^. However, the ECF regimen requires long hospitalisation rendering the application of this treatment difficult. The EOX regimen is more convenient than the ECF for patients and is commonly used in China. In the current study, we find that the administration approach to chemotherapy is one of the most important clinical predicator of pathologic tumor response. Regional arterial infusion chemotherapy can improve the pathological response rate of advanced gastric cancer with short-term EOX regimen NAC. Most of cytotoxic drugs have a steep dose-dependent response curve. Local interventional injection of concentration-dependent drug through artery improved its blood concentration^26^. Higher local drug concentrations would cause apoptosis and induce increased response rates^27^,^28^.

Chemotherapeutic response is also an important factor affecting the results. In the current study, improved RFS rates and OS rates were observed in the TRG3–4 cases. These findings were consistent with previous studies^29^,^30^. However, the influence of preoperative regional arterial infusion chemotherapy on the long-term survival was not clear. Zhang CW *et al*. has reported that preoperative regional arterial infusion chemotherapy plays an important role in improving the prognosis of advanced gastric cancer^11^. In the present study, improved RFS rates were observed in the regional arterial infusion group. However, no significant difference was observed in OS.

At the doses required for clinical efficacy, chemotherapeutic agents are often associated with significant side effects. Thus, a system that enables the delivery of chemotherapeutic compounds specifically to the organ of interest while reducing systemic exposure offers significant advantages. Regional arterial infusion chemotherapy offers this type of delivery system^28^,^31^,^32^. In this study, most bone marrow toxicities, vomiting, nausea, alopecia, and fatigue were slightly higher in the intravenous chemotherapy group. The chemotherapy-related toxicities decreased when patients were treated with regional arterial infusion chemotherapy compared to intravenous chemotherapy. On the other hand, surgical morbidities were not frequently observed in tow groups. Moreover, grade 3/4 complications were rare. No surgical mortality was observed.

In conclusion, we found that tumor differentiation, abnormal a-fetoprotein levels and administration approach to chemotherapy were significantly associated with pathological response. Regional arterial infusion NAC can improve the pathological response rate of advanced gastric cancer treated with EOX regimen.

This study is limited by its retrospective design and by the small nonrandomised group of patients who received regional arterial infusion NAC. Despite these limitations, our results are of interest because there is a lack of prospectively collected data on this topic in the current literature.

## Additional Information

**How to cite this article**: Wu, Z.-F. *et al*. Regional Arterial Infusion Chemotherapy improves the Pathological Response rate for advanced gastric cancer with Short-term Neoadjuvant Chemotherapy. *Sci. Rep.* **5**, 17516; doi: 10.1038/srep17516 (2015).

## Figures and Tables

**Table 1:** Univariate and Multivariate Analysis to Identify Predictors of Tumor Regression in Patients with Neoadjuvant Chemotherapy.

**Variables**	**Univariate**	**analysis**		**Multivariate analyses**		
	**Grade3and4**	**Grade0-2**	**P**	**HR**	**95% CI**	**P**
Gender			0.553	NA	NA	NA
Female	22	26				
Male	60	70				
Age			0.620	NA	NA	NA
≤60	60	66				
>60	22	30				
BMI			0.195	NA	NA	NA
≤20	13	23				
>20	69	73				
Anemia			0.603	NA	NA	NA
Yes	22	22				
No	60	74				
CEA(ng/ml)		0.195	NA		NA	NA
≤20	74	80				
>20	8	16				
CA72-4(U/ml)		0.302	NA		NA	NA
≤6	72	78				
>6	10	18				
AFP(ng/ml)		0.001		0.134	0.029 ~ 0.616	0.010
≤20	80	79				
>20	2	17				
CA19-9(U/ml)		0.826		NA	NA	NA
≤37	70	84				
>37	12	12				
Tumor location			0.740	NA	NA	NA
Upper body	20	25				
Middle body	23	27				
Lower body	28	36				
Diffuse type	11	8				
Tumor differentiation			0.002	0.404	0.203 ~ 0.792	0.008
Differentiated	63	52				
Undifferentiated	19	44				
Borrmann type			0.071	NA	NA	NA
I	18	11				
II	32	38				
III	21	39				
IV	11	8				
Clinical T classification			0.296	NA	NA	NA
cT2	23	34				
cT3	34	42				
cT4	25	20				
Clinical N classification			0.010	NA	NA	NA
cN−	33	58				
cN+			0.016	2.148	1.138 ~ 4.053	0.018
Preoperative Chemotherapy	49	38				
Intravenous chemotherapy	36	60				
Arterial infusion chemotherapy	46	36				

BMI body mass index, CEA carcinoembryonic antigen, CA72-4 carbohydrate antigen72-4, CA19-9 carbohydrate antigen 19-9, AFP alphafetoprotein.

**Table 2:** Adverse events by neoadjuvant chemotherapy.

	**Intravenous chemotherapy Group (n = 96)**		**Arterial infusion chemotherapy Group (n = 82)**	
	**All grades**	**Grade 3/4**	**All grades**	**Grade 3/4**
Leucocytes	64	3	41	1
Neutrophils	59	2	38	1
Hemoglobin	41	0	26	0
Platelets	32	0	22	0
Bilirubin	16	0	12	0
AST	22	0	19	0
ALT	24	0	13	0
Creatinine	13	0	7	0
Nausea	87	6	69	4
Vomitting	84	6	65	4
Anorexia	90	8	68	3
Mucositis	18	0	8	0
Diahhrea	16	1	10	0
Fatigue	72	4	53	1

**Table 3:** Surgical morbidity.

	**Intravenous chemotherapy Group**		**Arterial infusion chemotherapy Group**	
	**All grades**	**Grade 3/4**	**All grades**	**Grade 3/4**
Postoperative bleeding	1	0	2	1
Anastomotic leakage	2	2	2	1
Pancreas fistula	2	1	1	0
Abdominal abscess	1	0	0	0
Wound infection	0	0	1	0
Ileus	1	0	0	0
Pneumonia	2	0	0	0

**Table 4:** Comparison of the surgical and clinical pathological factors between intravenous chemotherapy group and arterial infusion chemotherapy group.

**Variables**	**intravenous chemotherapy group**	**arterial infusion chemotherapy group**	**P value**
Age			0.559
≤60	68	58	
>60	28	24	
BMI			0.511
≤20	19	17	
>20	77	65	
AFP(ng/ml)			0.453
≤20	85	74	
>20	11	8	
Tumor differentiation			0.316
Differentiated	60	55	
Undifferentiated	36	27	
Borrmann type			0.100
I	12	17	
II	45	25	
III	28	32	
IV	11	8	
Lauren type			0.176
Nonintestinal	19	11	
Intestinal	77	71	
Pathological T classification			0.967
T1	11	11	
T2	32	25	
T3	37	30	
T4	15	13	
Pathological N classification			0.991
N0	47	40	
N1	24	20	
N2	16	15	
N3	9	7	
Tumor regression grade			0.136
G0	7	5	
G1	21	11	
G2	32	20	
G3	35	43	
G4	1	3	
Type of surgery			0.309
Distal gastrectomy	41	39	
Total gastrectomy	55	43	
Combined with			0.476
splenectomy	19	15	
Yes	77	67	
No			

BMI body mass index, AFP alphafetoprotein.
